# SCON—a Short Conditional intrON for conditional knockout with one-step zygote injection

**DOI:** 10.1038/s12276-022-00891-0

**Published:** 2022-12-09

**Authors:** Szu-Hsien Sam Wu, Heetak Lee, Réka Szép-Bakonyi, Gabriele Colozza, Ayse Boese, Krista R. Gert, Natalia Hallay, Ji-Hyun Lee, Jihoon Kim, Yi Zhu, Margot M. Linssen, Sandra Pilat-Carotta, Peter Hohenstein, Hans-Christian Theussl, Andrea Pauli, Bon-Kyoung Koo

**Affiliations:** 1 Institute of Molecular Biotechnology of the Austrian Academy of Sciences (IMBA), Vienna BioCenter (VBC), Dr. Bohr-Gasse 3, 1030 Vienna, Austria; 2 Vienna BioCenter PhD Program, Doctoral School of the University at University of Vienna and Medical University of Vienna, 1030 Vienna, Austria; 3 Center for Genome Engineering, Institute for Basic Science, Expo-ro 55, Yuseong-gu, Daejeon, 34126 Republic of Korea; 4 Research Institute of Molecular Pathology (IMP), Vienna BioCenter (VBC), Campus-Vienna-BioCenter 1, 1030 Vienna, Austria; 5 Department of Medical and Biological Sciences, Catholic University of Korea, Bucheon, 14662 South Korea; 6 Transgenic Facility Leiden, Central Animal Facility, Leiden University Medical Center, Postbus 9600, 2300 RC Leiden, The Netherlands; 7 IMP/IMBA Transgenic Service, Institute of Molecular Pathology (IMP), Vienna, Austria

**Keywords:** Genetic engineering, Gene targeting, Genetic models

## Abstract

The generation of conditional alleles using CRISPR technology is still challenging. Here, we introduce a Short Conditional intrON (SCON, 189 bp) that enables the rapid generation of conditional alleles via one-step zygote injection. In this study, a total of 13 SCON mouse lines were successfully generated by 2 different laboratories. SCON has conditional intronic functions in various vertebrate species, and its target insertion is as simple as CRISPR/Cas9-mediated gene tagging.

## Intronic Genetics: Crisp control to switch off genes

In a procedure applicable to many different genes, the celebrated gene-editing technology known as CRISPR can insert a drug-controlled intronic ‘off switch’ into selected genes in mice via one-step zygote injections, allowing fine-tuned investigation of gene function. A research team led by Szu-Hsien Sam Wu and Bon-Kyoung Koo developed the procedure to generate intron-based conditional knock-out alleles. Here, the genetic modification is achieved using CRISPR gene-editing to insert a Short Conditional intrON called SCON (189 bp) into exons of genes. The authors successfully inserted SCON into 13 different genes. SCON is well tolerated in vivo when inserted into essential genes and fully compatible with the available recombinase lines for controlled switching off the gene when and where desired. SCON also shows potential application to other vertebrate species where zygote injection is feasible.

## Introduction

CRISPR gene editing has facilitated the investigation of gene function by precise gene knockout or knock-in. Upon the induction of gRNA-directed double-strand breaks (DSBs), the preferred repair pathway, nonhomologous end-joining (NHEJ), often leads to random insertions or deletions (indels), where out-of-frame mutations can cause partial or complete loss of gene function. On the other hand, by using a DNA template, DSBs can also be repaired via homology-directed repair (HDR), which allows the precise knock-in of various sequences into target loci. Due to the versatility and wide applicability of the CRISPR/Cas9 system, it has been utilized in numerous cell lines and lab organisms, across all biological and biomedical research fields^1^.

Despite the revolutionary advancement of CRISPR technology, the generation of conditional alleles has not been as easy as that of knockout or knock-in alleles. A conditional knockout (cKO) approach is often required to study essential genes such as housekeeping or developmentally required genes, as it allows the spatiotemporal control of gene knockout, thereby avoiding the early lethality associated with simple knockout. The use of cKO in rodents and eventually in nonhuman primates will therefore contribute to improved animal welfare. For many years, the Cre/loxP system has been widely utilized to construct cKO alleles by inserting two loxP recombination sites into the introns flanking essential exon(s). The generation of such “floxed” alleles in mice has traditionally involved the use of mouse embryonic stem (ES) cells, which are microinjected into blastocysts to generate chimeric mouse embryos^2^. Recently, cKO alleles have also been generated via the CRISPR/Cas9-mediated insertion of loxP sites in zygotes. However, this approach has turned out to be rather challenging, even with additional refinements^3^.

Here, we introduce the use of a universal conditional intron system for cKO approaches suitable for various animal models. Such a conditional intron approach has been attempted in the past, as it enables simple insertional mutagenesis with a fixed universal conditional intronic cassette^4–6^, but it has not been widely utilized in animal models because the cassettes were either too long^4,5^ or led to unexpected hypomorphic effects^6^. Notably, the simplest form currently is Degradation based on Cre-regulated-Artificial Intron (DECAI)^6^. Although the short size (201 bp) of the construct employed in this approach makes it desirable for gene targeting via zygote injection, it reduces target gene expression, compromising normal gene expression and animal development. Here, we present a fully optimized Short Conditional intrON (SCON) cassette that shows no hypomorphic effects in various vertebrate species and is suitable for targeting via one-step zygote microinjection.

## Materials and methods

### Mice

All animal experiments were performed according to the guidelines of the Austrian Animal Experiments Act, with valid project licenses approved by the Austrian Federal Ministry of Education, Science and Research, and were monitored by the institutional IMBA Ethics and Biosafety department.

#### Generation of SCON mice

*Ctnnb1-SCON* (*Ctnnb1*^*scon*^) cKO mice were generated via microinjection at the 2-cell stage, *Cdh12-SCON* (*Cdh12*^*scon*^) was generated via electroporation at the zygote stage, and the remaining 11 SCON lines (*Sox2*^*scon*^, *Lpar2*^*scon*^, *Mlh1*^*scon*^, *Ace2*^*scon*^, *Usp42*^*scon*^, *Sav1*^*scon*^, *Rnf34*^*scon*^, *Abi3*^*scon*^, *Lpar1*^*scon*^, *Tert*^*scon*^, and *Zmpste24*^*scon*^) were generated via microinjection at the 1-cell stage. For microinjection, we prepared 25 μl of CRISPR injection mix in nuclease-free buffer (10 mM TRIS-HCl, pH 7.4 and 0.25 mM EDTA), consisting of spCas9 mRNA (100 ng/μl), spCas9 protein (50 ng/μl), sgRNA (50 ng/μl), and ssODN (20 ng/μl, GenScript). The mixture was spun down in a tabletop centrifuge at 13,000 × *g* at 4 °C for 15–20 min to prevent the clogging of the injection needles. Frozen 2-cell-stage embryos of the C57Bl/6JRj background (JANVIER LABS) were used for cytoplasmic injection. For electroporation, a mix of components was prepared in Opti-MEM (Gibco; 31985062) including ctRNA 100 ng/μl (TracrRNA and crRNA annealed in IDT duplex buffer), SpCas9V3 100 ng/μl and ssODN 50 ng/μl (IDT technologies). Freshly obtained fertilized zygotes were added to the mix and electroporation was performed using NEPA21 (NEPAGENE) in a CUY501P1-1.5 slide, with the following settings: Poring pulse (40 V, pulse duration 3 ms, pulse interval 50 ms, 4 pulses) and transfer pulse (5 V, 50 ms, 50 ms, 5 pulses).

#### Tamoxifen administration and organ harvesting

*Ctnnb1*^*scon*^ mice were crossed with *Vil-CreER*^*T2*^ mice (B6. Cg-Tg(Vil1-cre/ERT2)23Syr/J, JAX, 020282)^7^ and bred to obtain either HET (*Vil-CreER*^*T2*^*; Ctnnb1*^*+/scon*^) or HOM (*Vil-CreER*^*T2*^*; Ctnnb1*^*scon/scon*^) mice. Tamoxifen (Sigma, T5648) dissolved in corn oil (Sigma, C8267) or corn oil alone was injected intraperitoneally into 8- to 12-week-old mice at a final concentration of 3 mg tamoxifen per 20 g body weight. Experiments were carried out in 2 mice of the respective genotype for each time point. Control mice received a matched volume of corn oil. On Day 3 or Day 5, mice were euthanized by cervical dislocation, and the intestines were harvested. The intestines were immediately cleaned with 1x PBS, flushed gently with 10% formalin solution (Sigma, HT501128), and fixed as ‘swiss rolls’ for 24 h at room temperature. The fixed intestines were washed three times with 1x PBS, with 2–3 h between each wash, before further processing.

### Genotyping

Toe clips or ear notches from the offspring were lysed in 30 μl of DirectPCR Lysis Reagent (Viagen) with 1 μl of proteinase-K (20 mg/ml; Promega, MC5005) at 55 °C overnight. The resulting mixture was diluted with 270 μl of nuclease-free water and spun down for at least 5 min in a tabletop centrifuge at 13,000 × *g*. Then, 2–3 μl of the clear part of the solution was used for PCR with either Gotaq (Promega, M7808) or LongAmp 2X (NEB, M0287S) according to the manufacturer’s instructions. To check the sequences of genomic DNA, PCR bands of the expected sizes were purified with a purification column.

### eGFP-SCON/eGFP-DECAI constructs

The eG-SCON-FP and eG-DECAI-FP cassettes were ordered from and synthesized by Genscript and subsequently cloned into the pcDNA4TO construct after digestion with BamHI (R0136S, NEB) and XhoI (R0146S, NEB) and ligation with T4 ligase (M0202S, NEB). The vectors were recombined with Cre-expressing bacteria (A111, Gene bridges) to obtain the recombined forms. The correct clones were confirmed by restriction digestion with SalI (R0138S, NEB) and Sanger sequencing.

### SCON A-stretch variants inserted into eGFP cDNA

eGFP cDNA containing SapI recognition sites at the selected intron insertion site was ordered from and synthesized by Genscript and cloned into the pcDNA4TO construct with BamHI and XhoI. Different SCON variant fragments containing the respective complementary ends were then inserted into eGFP with SapI (R0569S, NEB) and T4 ligase during 20 cycles of 2 min at 37 °C and 5 min at 16 °C, followed by 15 min at 37 °C and 10 min at 80 °C incubations^5^. The mixture was transformed into *Escherichia coli*, and DNA was extracted from individual colonies and checked with restriction digestion and Sanger sequencing.

### Cell culture and transfection

#### HEK 293T cells

Human embryonic kidney (Hek) 293T cells were cultured in high-glucose DMEM containing 10% fetal bovine serum (FBS, Sigma), 1% penicillin‒streptomycin (P/S; Sigma, P0781), and 1% L-glutamine (L-glut; Gibco, 25030024).

#### Mouse ES cells

The mouse ES cell line AN3-12 was cultured as previously described^8^ in high-glucose DMEM (Sigma, D1152) containing 10% FBS (Sigma), 1% P/S, 1% L-glut, 1% NEAA (Sigma, M7145), 1% sodium pyruvate (Sigma, S8636), 0.1 mM 2-mercaptoethanol (Sigma, M7522), and 30 μg/ml of mouse LIF (stock concentration: 2 mg/ml).

#### Cell lines of other species

The following cell lines were cultured with basal medium supplemented with 10% FBS and 1% P/S. The basal medium for each cell line is indicated in parentheses: C6 (ATCC, CCL-107; DMEM-F12 (Gibco, 31330038)), PK15 (Elabscience Biotechnology, EP-CL-0187; Minimal essential medium (Gibco, 11095080)), LLC-MK2 (Elabscience Biotechnology, ELSEP-CL-0141-1; RPMI-1640 (Sigma, R8758)), Vero (ATCC, CCL-81; DMEM-High glucose (Sigma, D1152)).

#### Plasmid transfection

A total of 500,000–750,000 cells were seeded into 6-well plates and allowed to attach and grow overnight. A total of 2.5 μg of DNA (1 μg of mCherry-expressing plasmid (Addgene, 72264) and 1.5 μg of pcDNA4TO-eGFP, -eG-SCON-FP, or -eG-DECAI-FP or recombined forms of -eG-SCON-FP or -eG-DECAI-FP) was mixed with 8 μl of polyethyleneimine (1 mg/ml; Polysciences, 23966), followed by incubation at room temperature for at least 15 min before being added dropwise to the cells. The culture medium was exchanged 8–10 h after transfection. Thirty-six hours after transfection, the cells were examined under an EVOS M7000 microscope (Thermo Scientific) with brightfield, GFP, and Texas Red filters. At 36–48 h after transfection, cells were dissociated into single cells for flow cytometry analysis with a BD-LSRFortessa flow cytometer (BD). Data from the flow cytometry experiments were analyzed in FlowJo software (BD). The fluorescence intensity values were exported and used for plotting and statistical tests using R.

### *Xenopus laevis* and *Danio rerio* embryo injection

*Xenopus laevis* eggs were collected and fertilized in vitro, as previously described^9^. A total of 100 pg of DNA, consisting of 50 pg of pcDNA4TO-eGFP, pcDNA4TO-eG-SCON-FP or pcDNA4TO-eG-SC-FP, and 50 pg of an mCherry-expressing plasmid, was injected into two dorso-animal blastomeres of *X. laevis* embryos at the 4-cell stage, and imaging was performed approximately 24 h later at the early tailbud stage. For *Danio rerio* experiments, 12 pg of DNA in total, consisting of 6 pg of pcDNA4TO-eGFP, pcDNA4TO-eG-SCON-FP or pcDNA4TO-eG-SC-FP, and 6 pg of mCherry-expressing plasmid, was injected into each fertilized egg/1-cell stage *D. rerio* embryo, and imaging was performed approximately 24 h later. *X. laevis* embryos were imaged using a stereomicroscope (Leica, M165MC) equipped with GFP (excitation: ET470/40 nm; emission: ET525/50 nm) and mCherry (excitation: ET560/40 nm; emission: ET630/75 nm) filters. *D. rerio* embryos were imaged with a Stereo Lumar.V12 fluorescence microscope (Zeiss) equipped with GFP (excitation 470/40 nm; emission 525/50 nm) and mCherry (excitation 550/25 nm; emission 605/70 nm) filters.

### Intestinal organoid culture

#### Establishment and maintenance

Crypts were isolated from the proximal part of the small intestine as reported previously^10^ and embedded in 15 μl droplets of BME-R1 (R&D Systems, 3433010R1) in a 48-well plate (Sigma, CLS3548-100EA). Organoids were established in WENR + Nic medium consisting of advanced DMEM/F12 (Gibco, 12634028) supplemented with penicillin/streptomycin (100x; Sigma, P0781), 10 mM HEPES (Gibco, 15630056), GlutaMAX (100x; Gibco, 35050061), B27 (50x; Life Technologies, 17504044), Wnt3 conditioned medium (Wnt3a L-cells, 50% of final volume), 50 ng/ml recombinant mouse epidermal growth factor (EGF; Gibco, PMG8041), 100 ng/ml recombinant murine Noggin (PeproTech, 250-38), R-spondin-1 conditioned medium (HA-R-Spondin1-Fc 293T cells, 10% of final volume) and 10 mM nicotinamide (Sigma, N0636). For the first week of culture, 100 μg/ml Primocin (InvivoGen, ant-pm-05) and 10 μM ROCK inhibitor/Y-27632 (Sigma, Y0503) were added to prevent microbial contamination and apoptosis, respectively. After the first passage, the established organoids were converted to ENR budding organoid cultures. Organoids were passaged at a ratio of 1:6 using mechanical dissociation.

#### 4-Hydroxytamoxifen treatment

Budding organoids at passage 3 or higher were passaged by mechanical dissociation and seeded in BME droplets. Medium containing vehicle (ethanol) or 500 nM 4-hydroxytamoxifen (Sigma, H7904) was added after BME polymerization. Eight hours later, the medium was exchanged back to ENR and replenished every two days.

### Histology and immunohistochemistry

Samples were processed using a standard tissue protocol on an Automatic Tissue Processor Donatello (Diapath). Samples were embedded in paraffin and cut into 2 μm sections on glass slides. Hematoxylin and eosin staining was carried out according to the standard protocol using a Gemini AS stainer (Thermo Scientific). For immunohistochemistry, the following antibodies were used: rabbit anti-Ki67 (1:200; 2 h at room temperature; Abcam, Ab16667) and rabbit anti-β-catenin (1:300; 1 h at room temperature; Abcam, ab32572). For signal detection, a two-step HRP-conjugated rabbit polymer system (DCS, PD000POL-K) was used. Stained slides were imaged with a 40x objective using the Pannoramic FLASH 250 III scanner (3DHISTECH), and images were cropped using CaseViewer software.

To identify *Olfm4* and *Wnt3* mRNAs, we performed an RNA in situ hybridization (ISH) assay using an RNAscope® Multiplex Fluorescent Detection Kit v2 (ACDBio 323110). The assay was performed according to the standard manual provided by the ACD company. Briefly, paraffin-embedded samples were freshly cut to a 4 μm thickness on glass slides one week prior to staining, and the slides were dried and stored only at room temperature. On the day before staining, the slides were manually deparaffinized, pretreated, and stored overnight at room temperature for further processing. Duplex staining was performed according to the manufacturer’s manual on the next day with the probes for *Olfm4* (311831-C2, Cy3) and *Wnt3* (312241, Cy5). Stained slides were imaged with a 40x objective using a Pannoramic FLASH 250 III scanner (3DHISTECH), and images were cropped using CaseViewer software.

### Immunofluorescence analysis of intestinal organoids

BME droplets containing organoids were carefully collected into 1.5 ml tubes and spun down in a tabletop centrifuge at 600 × *g* for 5 min. The supernatant and visible fraction of attached BME were removed. The pellet was resuspended in 4% paraformaldehyde and fixed at room temperature for 15–20 min. Fixed organoids were washed 3 times in 1x PBS at 10–15-min intervals. The organoids were blocked and permeabilized in a solution containing 5% DMSO, 0.5% Triton-X-100 (Sigma, T8787), and 2% normal donkey serum (Sigma, D9663) for one hour at 4 °C. The samples were stained overnight with Alexa 647-conjugated mouse anti-β-catenin (1:200; Cell Signaling Technology, 4627S) and ATTO 488-conjugated phalloidin (1:300; Sigma, 49409-10NMOL). The samples were washed three times with 1x PBS. During the last wash, the samples were incubated with 2 μg/ml DAPI before mounting on coverslips in a solution containing 60% glycerol and 2.5 M fructose^11^, followed by imaging on a multiphoton SP8 confocal microscope (Leica).

### Computing SCON-targetable sites

To construct databases of SCON insert sites, we used genomic information on sequences, exons, coding regions, and gene types derived from Ensembl Biomart, build 102 (http://www.ensembl.org/)^12^ for mouse (*M. musculus*), rat (*R. norvegicus*), macaque (*M. mulatta*), marmoset (*C. jacchus*), and medaka (*O. latipes*). To map canonical transcripts to genes, we derived ‘PRINCIPAL:1’ from the APPRIS database for mice and rats with Ensembl build 102 (http://appris-tools.org)^13^, and we regarded the longest transcript as a canonical transcript for other species. The quality features, including GC content, self-complementarity, and mismatch scores, of each candidate site were mapped by the same approach employed by CHOPCHOP (https://chopchop.cbu.uib.no)^14^. Of note, mismatch scores were calculated using Bowtie (v1.2.2)^15^ with the ‘-v 3 -a’ option with 23 bp target sequences. The codes will be made available from the corresponding authors upon request.

## Results

### SCON is a versatile conditional intron with no discernable hypomorphic effect

**Fig. 1:**
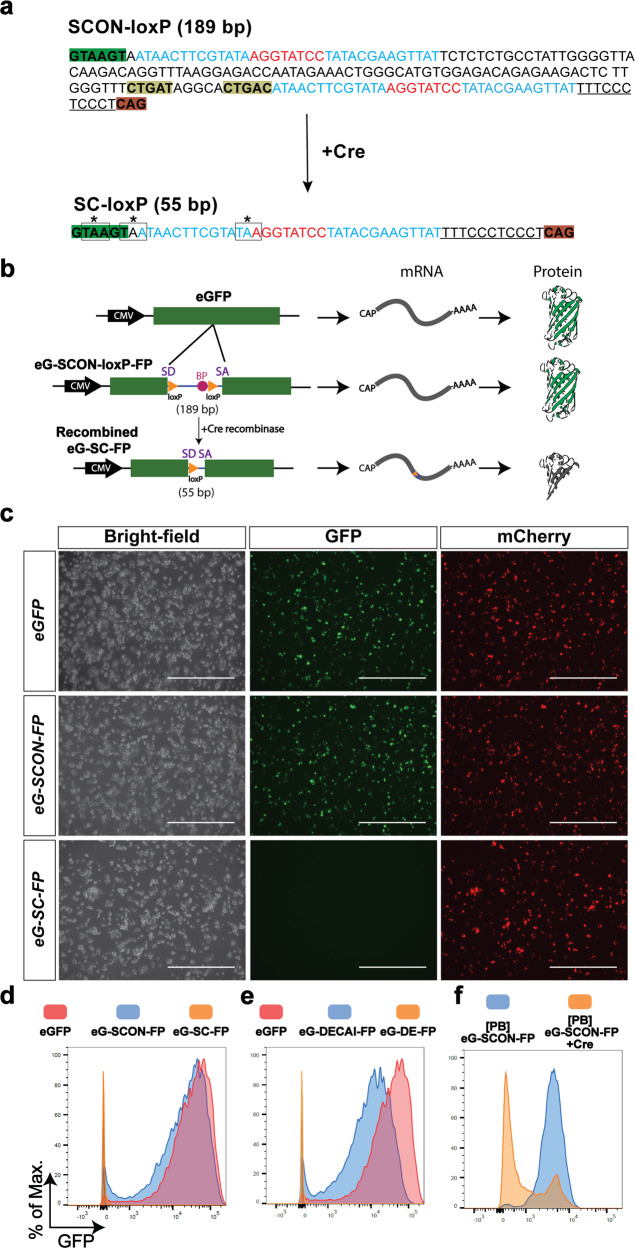
SCON is a novel cKO approach that shows desired intronic functionality in vitro. **a** Sequence of SCON before and after recombination. The intact SCON is 189 bp long, and the sequence is annotated as follows: nucleotides highlighted in green and dark orange at the two ends represent the splice donor and splice acceptor, respectively; nucleotides highlighted in dark yellow represent the putative branch points; sequences that are underlined represent the polypyrimidine tract; nucleotides in blue and red represent loxP sites consisting of 13 bp recognition sites and 8 bp spacers, respectively. In the recombined SCON form, which is 55 bp long, the remaining components include the splice donor, one loxP, polypyrimidine tract, and the splice acceptor; the three coding frames contain stop codons that are indicated by a box and an asterisk over the nucleotides. **b** Schematic diagram of the SCON functionality test in an eGFP overexpression construct including intact eGFP, eG-SCON-FP, and recombinant eG-SC-FP. SD, splice donor; BP, branch point; SA, splice acceptor. **c** Images of transfected HEK293T cells on Day 1 with intact eGFP, eG-SCON-FP and recombinant eG-SC-FP. All constructs were cotransfected with an mCherry overexpression plasmid. Scale bar, 1 mm. **d**, **e** Histograms of the flow cytometry analysis of transfected HEK293T cells showing comparisons between eGFP (red) and eG-SCON-FP (blue) (**d**), between eGFP (red) and eG-DECAI-FP (blue) (**e**), and the respective recombined forms eG-SC-FP and eG-DE-FP (yellow) (**d**, **e**). **f** Flow cytometry analysis of mouse ES cells with integrated piggyBac-eG-SCON-FP transfected with Cre-expressing plasmid (yellow) or empty vector (blue).

SCON is a modified intron derived from the first intron (130 bp) of the human *HBB* (hemoglobin subunit beta) gene. SCON is 189 bp long, consisting of (from the 5′ to 3′ end) a splice donor, a loxP recombination site, a branch point, a second loxP recombination site, a polypyrimidine tract, and a splice acceptor. By design, it has a similar sequence architecture to the previously introduced conditional intronic system DECAI (201 bp)^6^, which showed a hypomorphic effect at the level of protein expression. To optimize conditional intron function, several features were implemented in SCON: (1) the length between the putative branch point and the splice acceptor was kept to 45 bp to allow efficient splicing to take place, (2) the distance between the two loxP sites was 100 bp for efficient Cre-loxP recombination, and (3) miscellaneous changes were incorporated for optimal splice donor, acceptor, and pyrimidine tract sequences. Upon recombination, SCON is reduced to 55 bp, in which all three reading frames contain translational stop codons within the remaining loxP sequence, such that loss of gene function occurs via premature translational termination (Fig. 1a).

First, we validated the functionality of SCON in an eGFP overexpression construct. We cotransfected HEK293T cells with an mCherry cassette (serving as a transfection control) and a cassette containing either intact eGFP, eG-SCON-FP, or already Cre-recombined eG-SCON-FP (eG-SC-FP) (Fig. 1b) and assessed the resultant fluorescence intensity by fluorescence microscopy and flow cytometry (Fig. 1c, d). We also carried out the same test in parallel with DECAI for comparison. Both eG-SCON-FP and eG-DECAI-FP showed GFP expression, whereas the recombined form had no detectable fluorescence (Fig. 1c–e). Interestingly, eG-SCON-FP exhibited similar GFP signal levels as the intact eGFP construct (Fig. 1d). However, eG-DECAI-FP showed reduced levels (Fig. 1e), indicating an adverse hypomorphic effect as an intron, which is in line with previous observations^6^. Thus, our SCON showed a reliable conditional intronic function with no discernable hypomorphic effect.

Next, we tested whether SCON could be efficiently recombined in mammalian cells. We cloned the eG-SCON-FP construct into a piggyBac transposon backbone and generated clonal mouse ES cell lines with constitutive eGFP expression containing SCON. Cotransfecting a Cre-expressing plasmid with an mCherry cassette into these eG-SCON-FP-expressing ES cells resulted in efficient reduction of eGFP levels within the mCherry+ population, whereas the mock control (transfected only with the mCherry cassette) maintained high levels of eGFP (Fig. 1f). Taken together, our data indicate the suitability of SCON for use in mammalian systems, where it is neutral upon insertion and can be efficiently recombined by Cre recombinase to abolish its intronic function and cause knockout of the inserted gene.

**Fig. 2:**
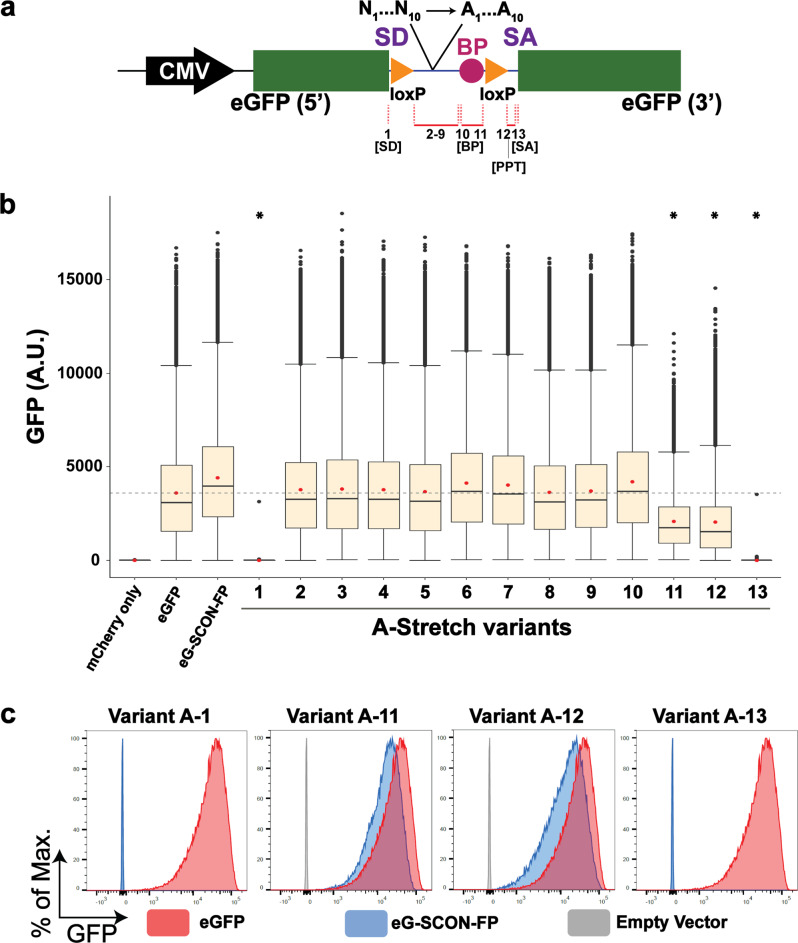
Dissection of functional sequences within the SCON cassette through A-stretch mutagenesis. **a** Schematic diagram of the 13 A-stretch variants, where 6–10 nucleotides are converted to adenine, covering all sequences from SD to SA, excluding the LoxP sites. PPT, polypyrimidine tract. **b** Boxplots of measured scaled values from the flow cytometry analysis of HEK293T cells transfected with mCherry alone, eGFP, eG-SCON-FP, and the 13 A-stretch variants. The red dot indicates the mean. The gray horizontal dotted line indicates the mean value of eGFP. *indicates statistical significance (*p* = 0) from the unpaired t test with negative mean difference values when compared with the mean intensity value of eGFP. **c** Flow cytometry analysis of HEK293T cells transfected with variant A-1, A-11, A-12, or A-13 (blue) compared with intact eGFP (red) and the empty vector or mCherry alone (gray).

To better understand the functionality of different components within the SCON cassette, we designed a series of “A-stretch variants” in which 6–10 nt within SCON was converted to adenine (Fig. 2a). Among the 13 different variants, we found that four showed either hypomorphic or a complete loss of eGFP fluorescence when inserted as an intron (Fig. 2b, c). These included the branch point (variant 11) and the polypyrimidine tract (variant 12), which resulted in hypomorphic expression of the inserted eGFP cassettes, whereas the splice donor (variant 1) and the splice acceptor (variant 13) resulted in a complete loss of eGFP expression (Fig. 2b, c). These data suggest that all elements for splicing are required for the optimal intronic function of SCON, while most components between the two loxP sites are less essential.

### The neutrality of SCON is conserved in various vertebrate species

**Fig. 3:**
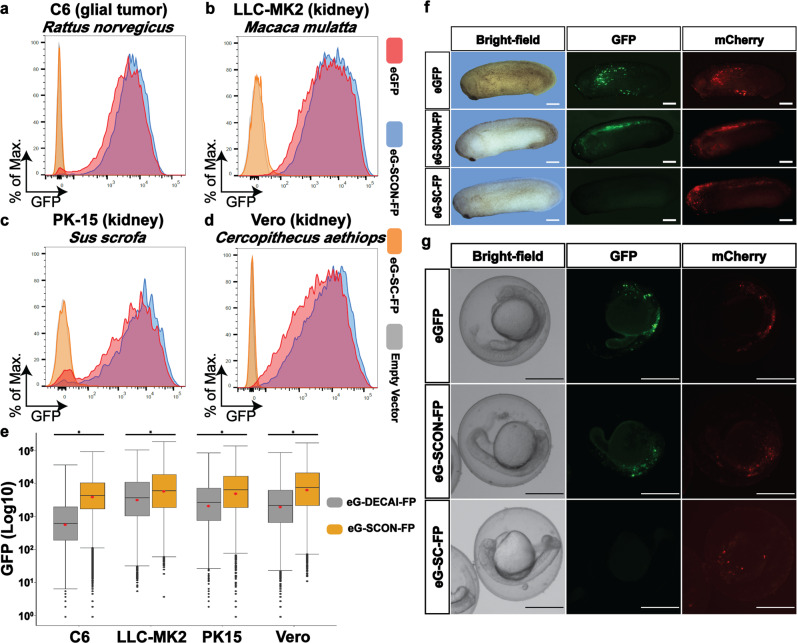
SCON is applicable and neutral in various vertebrate species. **a**–**d** Flow cytometry analysis of C6 (**a**), LLC-MK2 (**b**), PK-15 (**c**), and Vero (**d**) cells transfected with eGFP (red), eG-SCON-FP (blue), eG-SC-FP (yellow) or the empty vector or mCherry alone (gray). **e** Flow cytometry analysis comparing the GFP levels in various cell lines transfected with eG-DECAI-FP (gray) or eG-SCON-FP (yellow). *p < 0.001, from unpaired t test. **f**, **g** *Xenopus* and zebrafish embryos injected with eGFP, eG-SCON-FP, or eG-SC-FP constructs, which were imaged 24 h postinjection. A plasmid containing mCherry was used as an injection control. Scale bar, 500 μm.

cKO alleles have been widely used in mice for decades because of the robust methodology involving germline-competent ES cells^16^, the endeavors of the International Knockout Mouse Consortium^17^, and, recently, the advent of CRISPR technology^18^. However, in many other vertebrate species, such as zebrafish, frog, rat, pig, cattle, and nonhuman primate models, the application of the cKO approach has been limited, mainly due to the lack of reliable germline-competent ES cells. We therefore sought to test whether SCON may be a suitable cKO strategy for non-murine species. To this end, we used cell lines from different species, including C6 (*Rattus norvegicus*), PK15 (*Sus scrofa*), LLC-MK2 (*Macca mulatta*), and Vero (*Cercopithecus aethiops*) cells, and transfected them with eGFP, eG-SCON-FP or eG-DECAI-FP overexpression constructs and the corresponding Cre-recombined forms. In line with the results in HEK293T and mouse ES cells, the SCON intron did not cause any discernable hypomorphic effects in any of the cell lines tested (Fig. 3a–d, in blue compared with red), while the recombined forms of SCON abrogated eGFP expression completely (Fig. 3a–d, in orange). Intriguingly, in three of the cell lines (C6, PK15, and Vero), eG-DECAI-FP showed strikingly reduced levels of eGFP compared to eG-SCON-FP (Fig. 3e), again confirming that SCON is devoid of the hypomorphic effects associated with DECAI. In addition, we also explored the possibility of utilizing SCON in frogs (*Xenopus laevis*) and zebrafish (*Danio rerio*) by injecting eGFP, eG-SCON-FP, and Cre-recombined plasmids into 4-cell-stage embryos or fertilized eggs, respectively. We examined the embryos 24 h postinjection. and observed that eGFP and eG-SCON-FP expressed readily detectable eGFP fluorescence, whereas the embryos that received the recombined form did not (Fig. 3f, g). Together, these data indicate that SCON has the potential to be utilized as a cKO system in a wide variety of vertebrate species.

### Targeted insertion of SCON via one-step zygote injection

**Fig. 4:**
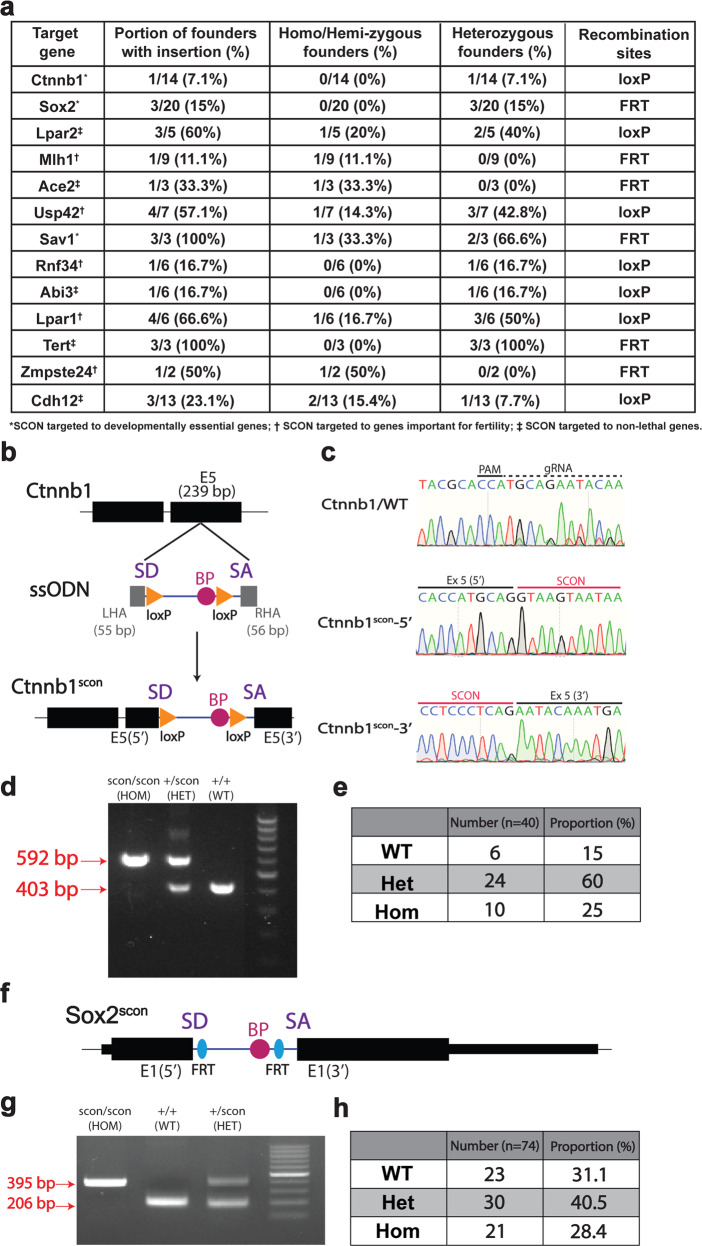
SCON mice can be generated via one-step embryo injection, and SCON is tolerated in homozygosity. **a** List of alleles successfully targeted with SCON and the corresponding efficiencies, genotypes, and recombination sites used. **b** Schematic illustration of SCON targeting ssODN, with 55 and 56 bp left and right homology arms, respectively, in exon 5 of the *Ctnnb1* gene. **c** Sanger sequencing track of the WT, 5′ and 3′ alleles of *Ctnnb1* and *Ctnnb1*^*scon*^, respectively. **d** Genotyping PCR of *Ctnnb1*^*scon/scon*^ (HOM), *Ctnnb1*^*+/scon*^ (HET), and *Ctnnb1*^*+/+*^ (WT), in which the lower (403 bp) and upper (592 bp) bands correspond to the WT and knock-in alleles, respectively. **e** Genotype quantification from crosses of double heterozygotes (*Ctnnb1*^*+/scon*^). Total number of offspring, *n* = 40. **f** Schematic illustration of the *Sox2*^*scon*^ allele. **g** Genotyping PCR of *Sox2*^*scon/scon*^ (HOM), *Sox2*^*+/+*^ (WT), and *Sox2*^*+/scon*^ (HET), in which the lower (206 bp) and upper (395 bp) bands correspond to the WT and knock-in alleles, respectively. **h** Genotyping quantification from crosses of heterozygotes (*Sox2*^*+/scon*^). Total number of offspring, *n* = 74.

As our results from the in vitro overexpression-based systems demonstrated the suitability of SCON for cKO approaches, we next sought to test whether it would also work well for targeting endogenous genes in vivo. Therefore, we chose to generate a SCON cKO *Ctnnb1* allele (Fig. 4a, b), which encodes β-catenin and is a developmentally required gene whose knockout causes early lethality in mice. We injected CRISPR ribonucleoprotein (RNP) with 300 bp-long, commercially synthesized, single-stranded deoxynucleotides (ssODNs) consisting of SCON with short homology arms (55 and 56 bp for the 5′ and 3′ homology arms, respectively) into the cytoplasm of developing 2-cell stage mouse embryos^19^. Genotyping and sequencing revealed that one out of the resulting 14 offspring (7.1%) showed precise heterozygous integration, with the other allele remaining intact (Fig. 4c). From these offspring, we were able to perform backcrossing to confirm germline transmission, and we continued breeding until achieving homozygosity (*Ctnnb1*^scon/scon^) (Fig. 4d). From the heterozygote-to-heterozygote (HET) crosses, we did not observe underrepresented ratios of homozygous (HOM) mice (Fig. 4e), and the HOM mice showed no discernible phenotype, confirming intact gene function with SCON insertion. Similarly, we generated a second SCON cKO *Sox2* allele with FRT recombination sites (Fig. 4f). Sox2 mice appeared normal and could be bred to homozygosity (Fig. 4g), and HET-HET crosses yielded the expected proportion of HOM mice (Fig. 4h). In the current study, we successfully inserted SCON into 13 different alleles in mice across two different laboratories (Fig. 4a), which are at different stages of breeding to confirm proper functionality. These results again indicate that SCON is suitable for generating cKO alleles in vivo.

**Fig. 5:**
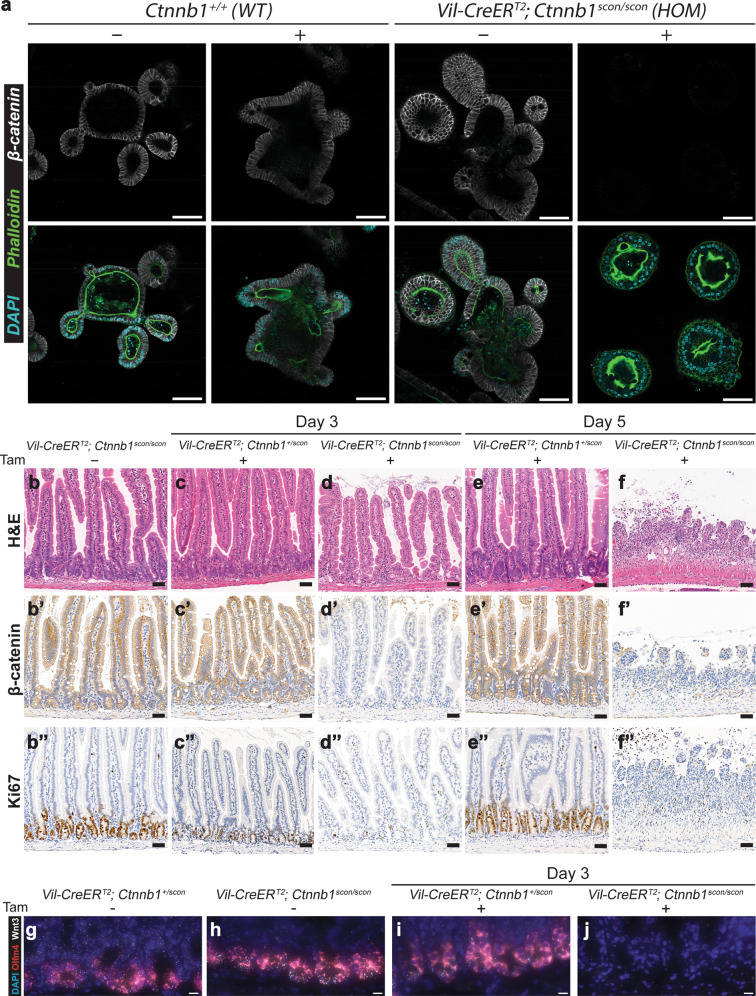
*Ctnnb1*^*scon*^ mice harbor a functional conditional allele that functions in vivo. **a** Small intestinal organoids from WT (*Ctnnb1*^*+/+*^) and HOM (*Vil-CreER*^*T2*^*; Ctnnb1*^*scon/scon*^) mice treated with either 4-OH-tamoxifen (4-OHT) or vehicle for 8 h. Organoids were fixed on Day 4 and stained for β-catenin (gray), phalloidin (green) and DAPI (cyan). Scale bar, 100 μm. Homozygous (*Vil-CreER*^*T2*^*; Ctnnb1*^*scon/scon*^) intestines are healthy, with a normal epithelial crypt-villus morphology (**b**), β-catenin on the cell membrane (**b**′), and Ki67 marking proliferating cells in the crypts (**b**″). Heterozygous (*Vil-CreER*^*T2*^*; Ctnnb1*^*+/scon*^) intestines show no changes in morphology (**c**, **e**), β-catenin (**c**′, **e**) or Ki67 (**c**″, **e**″) after tamoxifen treatment. In homozygous intestines, tamoxifen treatment leads to the loss of crypts (**d**), β-catenin staining (**d**′), and Ki67 staining (**d**″) on Day 3. On Day 5, the epithelium is completely lost (**f**–**f**″). H&E, hematoxylin and eosin. Scale bar, 50 μm. **g**–**j** smRNA-FISH of intestinal sections with DAPI (blue), *Olfm4* (red), and *Wnt3* (white). **g**, **h** uninduced intestinal sections of HET (**g**) and HOM (**h**) mice; **i**, **j** Day 3 after induction with 3 mg tamoxifen per 20 g body weight of HET (**i**) and HOM (**j**) mice. Scale bar, 10 μm.

To verify the conditional functionality of the *Ctnnb1*^*scon*^ allele, we utilized *Villin-CreER*^*T2*^ (*Vil-CreER*^*T2*^) for intestinal epithelium-specific Cre recombination. We first isolated crypts from the duodenum of HOM (*Vil-CreER*^*T2*^; *Ctnnb1*^scon/scon^) and wild-type (*Ctnnb1*^+/+^) mice to establish adult stem cell-based intestinal organoids. Then, budding organoid cultures (with EGF, Noggin, and R-spondin1) were transiently treated with 4-OH-tamoxifen for 8 h. Both 4-OH-tamoxifen-treated and untreated wild-type organoids as well as untreated HOM organoids continued to grow normally (Fig. 5a). However, 4-OH-tamoxifen-treated HOM organoids ceased growth or collapsed from Day 2 onward. Immunolabeling confirmed the loss of β-catenin in the small cystic mutant organoids, while DAPI and phalloidin staining showed that the cells remained alive (Fig. 5a).

To directly verify functionality in vivo, we injected 3 mg tamoxifen per 20 g body weight into both HOM (*Vil-CreER*^*T2*^; *Ctnnb1*^scon/scon^) and HET (*Vil-CreER*^*T2*^; *Ctnnb1*^+/scon^) mice and harvested the intestines on Day 3 and Day 5. Control samples showed a normal crypt-villus axis, detectable membrane-bound β-catenin, and a Ki67+ proliferative zone at the bottom of the crypts, where stem and progenitor cells are located (Fig. 5b, b′, b″, c, c′, c″, e, e′, e″). On Day 3, tamoxifen-treated HOM samples showed a clear loss of proliferative crypts with loss of β-catenin staining (Fig. 5d, d′, d″). On Day 5, tamoxifen-treated HOM mice exhibited shortened and inflamed intestines, sections of which showed nearly complete loss of intestinal epithelium (Fig. 5f, f′, f″). These data indicate efficient recombination of the *Ctnnb1*^scon^ allele in vivo and the loss of β-catenin function upon recombination. We also used single-molecule RNA fluorescent in situ hybridization (smRNA-FISH) for the analysis of *Olfm4* and *Wnt3*, which are a marker of intestinal stem cells and a marker of a molecule secreted by Paneth cells, respectively. Both *Olfm4* and *Wnt3* are expressed in uninduced crypts of both HET (Fig. 5g) and HOM (Fig. 5h) genotypes. On Day 3 post-tamoxifen injection, *Olfm4* and *Wnt3* expression remained in the HET crypts (Fig. 5i) but was lost in the HOM intestine (Fig. 5j), indicating the loss of stem cells and Paneth cells.

### A large fraction of protein-coding genes are SCONable

**Fig. 6:**
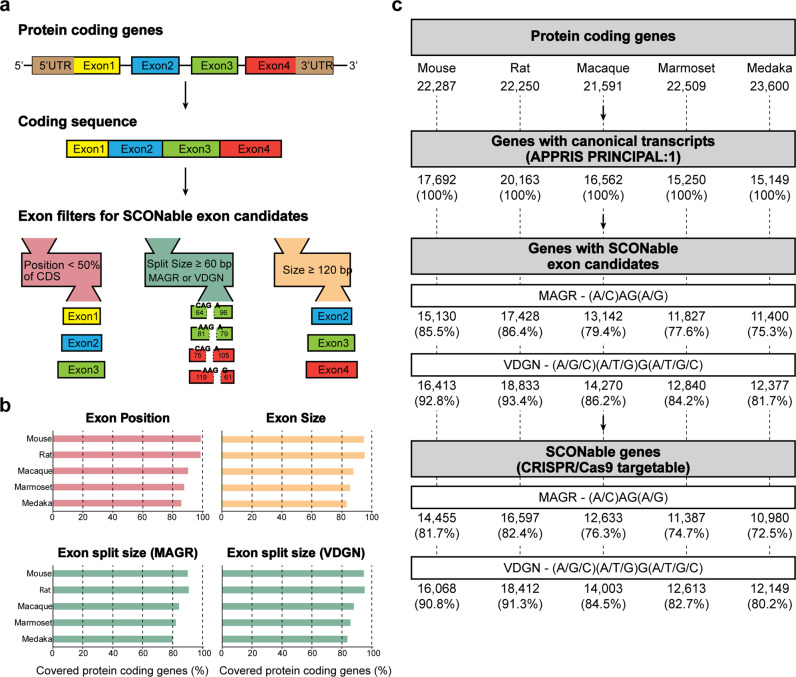
Scheme for the construction of the database of SCON insertion sites. **a** Selection of the target exon candidates from protein-coding transcripts. **b** Gene coverage of individual exon filters, such as position, size, and split exon size. **c** Summary of databases of SCON insertion sites with CRISPR/Cas9 target sites.

To systematically estimate targetable sites for SCON, we carried out bioinformatic analysis to screen for possible insertion sites in mouse, rat, macaque, marmoset and medaka genomes (Fig. 6). The selection criteria included the following: (1) target exons are positioned within the first 50% of the protein-coding sequence from canonical transcripts of protein-coding genes; (2) intron insertion sites contain either stringent (MAGR, (A/C)-A-G-(A/G))^20^ or flexible (VDGN, (A/G/C)-(A/T/G)-G-(A/T/G/C))^21^ splice junction consensus sequences; and (3) exons must be longer than 120 bp in length, where both 5′ and 3′ split exons must be at least 60 bp (Fig. 6a)^5^. After applying all selection criteria, we found that the majority of coding genes were targetable in all five species (80.8% for MAGR and 87.7% for VDGN intron insertion sites on average) (Fig. 6b, c). We also found CRISPR/Cas9 targeting site(s) around intron insertion sites in most cases (Fig. 6c and Supplementary Tables 1–10). This is a conservative estimate, as some genes with an important domain close to the 3′ end can still be targeted in the second half and, if necessary, a novel intron insertion site can be generated by introducing silent mutations.

## Discussion

**Fig. 7:**
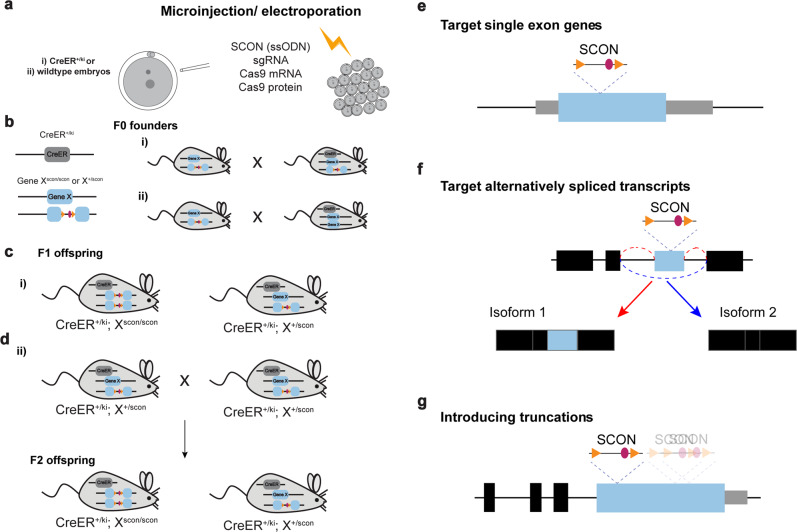
Scheme of SCON mouse generation in 1–2 crossing steps and specific target gene application. **a** SCON mouse generation in 1-cell-stage embryos via microinjection or electroporation. The embryos either (i) carry inducible CreER alleles or (ii) are wild-type. **b** The F0 founder mice are screened for SCON insertion in the target gene (gene X). Mice carrying a SCON insertion are crossed with each other (i) or are crossed with a CreER-bearing mouse (ii). **c** The resulting F1 offspring may contain genotypes ready for in vivo cKO studies (i). **d** The F1 offspring that carry a gene X-SCON allele as well as the CreER allele are crossed with each other to obtain experiment-ready cKO mice in the F2 generation (ii). **e** Illustration of the insertion of SCON in single-exon genes. **f** Illustration of the insertion of SCON into genes that express alternatively spliced transcripts. SCON can be inserted in the alternatively spliced exon, which would lead to the protein truncations produced by isoform 1, while isoform 2 is intact. **g** Illustration of the insertion of SCON in large exons (>1000 bp), which leads to various sizes of truncations.

In summary, our SCON-mediated cKO approach transforms the complicated process of generating conditional alleles into the simple, CRISPR-mediated knock-in of a 189 bp intronic sequence through one-step zygote injection. The complete generation of an experimental line with SCON requires 1–2 rounds of breeding. Microinjection or electroporation of targeting components (300 bp ssODN, sgRNA, and Cas9 protein and mRNA) was first performed in (i) zygotes carrying inducible CreER alleles or (ii) wild-type zygotes (Fig. 7a). Founder mice with precise SCON insertion into the target gene were selected for further breeding with each other or with another mouse carrying the CreER allele (Fig. 7b). As a result, (i) experimentally ready cKO and control mice can be obtained in the F1 generation (Fig. 7c), whereas in case (ii), homozygous SCON mice as well as CreER allele-carrying mice are obtained in the F2 generation (Fig. 7d).

SCON is superior to the conventional ‘floxed’ approach in targeting single-exon (intronless) genes (Fig. 7e), genes with various alternatively spliced isoforms (Fig. 7f) and large exons encoding different protein domains (Fig. 7g). Approximately 3% of genes in the human genome are single-exon genes. These include transcription factor genes, genes important for signal transduction and tissue-specific developmental regulator genes^22^. The SCONing approach for generating cKO model organisms would therefore be desirable for studying functions and diseases associated with single-exon genes. Alternative splicing plays an important role in development and can be heterogeneously regulated depending on the cell population^23^, as revealed by advanced single-cell RNA sequencing technologies^24^. Being able to conditionally deplete specific isoform(s) would be desirable for further understanding complex context-dependent gene regulation. Targeting the alternatively spliced exon(s) with SCON would, in theory, produce an inactive truncated isoform without compromising the original transcript level (Fig. 7f). Finally, genes containing large exons (>1 kb), such as *Tert*, *Brca1/2,* and *Apc*, often encode multiple protein domains. To achieve partial or complete domain truncations, SCON can be inserted at a suitable location to achieve particular truncations while maintaining the N-terminal fraction (Fig. 7g).

This novel strategy gives rise to the exciting possibility of generating conditional knockouts in other vertebrate models ranging from fish to nonhuman primates. A key to our success is the creation of a neutral artificial intron ready for Cre-mediated inactivation. In contrast to DECAI, which shows undesirable hypomorphic effects that likely limit its utility in cKO animal models, SCON is well tolerated and is ideal for the rapid generation of cKO animals. Moreover, loxP sequences can be replaced by other recombination sites (e.g., FRT) for the rapid generation of FRT- or other recombinase-based conditional alleles that are not yet widely utilized. Finally, the dispensable region between the two loxP sites serves as a harboring space for the addition of other genetic elements. We expect that the SCON strategy will become the foundation for a new cKO approach in biomedical and industrial research that is well-suited for addressing animal welfare concerns.

## Supplementary information

Supplementary materials

Dataset 1

Dataset 2

Dataset 3

Dataset 4

Dataset 5

Dataset 6

Dataset 7

Dataset 8

Dataset 9

Dataset 10
