# Author Correction: SCON—a Short Conditional intrON for conditional knockout with one-step zygote injection

**DOI:** 10.1038/s12276-023-01039-4

**Published:** 2023-06-21

**Authors:** Szu-Hsien Sam Wu, Heetak Lee, Réka Szép-Bakonyi, Gabriele Colozza, Ayse Boese, Krista R. Gert, Natalia Hallay, Ji-Hyun Lee, Jihoon Kim, Yi Zhu, Margot M. Linssen, Sandra Pilat-Carotta, Peter Hohenstein, Hans-Christian Theussl, Andrea Pauli, Bon-Kyoung Koo

**Affiliations:** 1 Institute of Molecular Biotechnology of the Austrian Academy of Sciences (IMBA), Vienna BioCenter (VBC), Dr. Bohr-Gasse 3, 1030 Vienna, Austria; 2 Vienna BioCenter PhD Program, Doctoral School of the University at University of Vienna and Medical University of Vienna, 1030 Vienna, Austria; 3 Center for Genome Engineering, Institute for Basic Science, Expo-ro 55, Yuseong-gu, Daejeon, 34126 Republic of Korea; 4 Research Institute of Molecular Pathology (IMP), Vienna BioCenter (VBC), Campus-Vienna-BioCenter 1, 1030 Vienna, Austria; 5 Department of Medical and Biological Sciences, Catholic University of Korea, Bucheon, 14662 South Korea; 6 Transgenic Facility Leiden, Central Animal Facility, Leiden University Medical Center, Postbus 9600, 2300 RC Leiden, The Netherlands; 7 IMP/IMBA Transgenic Service, Institute of Molecular Pathology (IMP), Vienna, Austria

**Keywords:** Genetic engineering, Gene targeting, Genetic models, Genetic engineering, Gene targeting, Genetic models

Correction to: *Experimental and Molecular Medicine* 10.1038/s12276-022-00891-0, published online 09 December 2022

After online publication of this article, the authors noticed an error in the Fig. 5 c’’ section.

The correct statement of this article should have read as below.

In Fig. 5 of the article, panels c’’ and e’’ were inadvertently cropped from the same captured image. The correct Fig. 5c’’ has been updated to show the intestine section stained with Ki67 from Vil-CreERT2; Ctnnb1+/scon animal on day 3 after tamoxifen injection. The correction of Fig. 5c’’ does not influence the conclusion of the in vivo functionality experiments in the Ctnnb1scon animals or the overall conclusion of the paper.
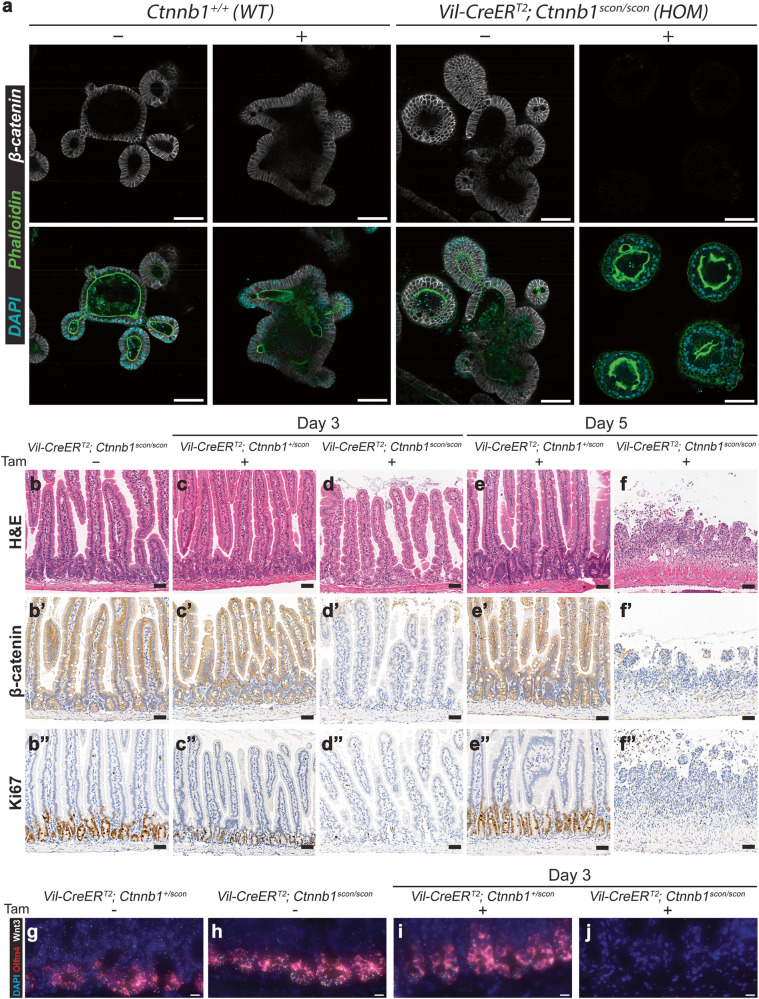


The authors apologize for any inconvenience caused.

The original article has been corrected.

