# Supplementary material for: SCON—a Short Conditional intrON for conditional knockout with one-step zygote injection
Source: Exp Mol Med. 2022 Dec 9;54(12):2188–99. doi: 10.1038/s12276-022-00891-0 (PMC9794761; doi:10.1038/s12276-022-00891-0)
Supplement: Supplementary file 1 — Supplementary materials [file 12276_2022_891_MOESM1_ESM.docx]

**Supplementary materials**

**gRNAs and repair templates used in SCON mouse generation**

**Ctnnb1**: TACATCATTTGTATTCTGCATGG

ACATGCCATCATGCGCTCCCCTCAGATGGTGTCTGCCATTGTACGCACCATGCAGGTAAGTAATAACTTCGTATAAGGTATCCTATACGAAGTTATTCTCTCTGCCTATTGGGGTTACAAGACAGGTTTAAGGAGACCAATAGAAACTGGGCATGTGGAGACAGAGAAGACTCTTGGGTTTCTGATAGGCACTGACATAACTTCGTATAAGGTATCCTATACGAAGTTATTTTCCCTCCCTCAGAATACAAATGATGTAGAGACAGCTCGTTGTACTGCTGGGACTCTGCACAACCTTTC

**Sox2**: TCTGCACATGAAGGAGCACCCGG

CGAGAAGCGGCCGTTCATCGACGAGGCCAAGCGGCTGCGCGCTCTGCACATGAAGGTAAGTAGAAGTTCCTATTCtctagaaaGtATAGGAACTTCTCTCTCTGCCTATTGGGGTTACAAGACAGGTTTAAGGAGACCAATAGAAACTGGGCATGTGGAGACAGAGAAGACTCTTGGGTTTCTGATAGGCACTGACGAAGTTCCTATTCtctagaaaGtATAGGAACTTCTTTCCCTCCCTCAGGAGCACCCGGATTATAAATACCGGCCGCGGCGGAAAACCAAGACGCTCATGAAGAA

**Lpar2**: ATGGAGAGCCTGGCAGTGCGTGG

TCGCTGGCATGGCCTACCTCTTCCTCATGTTCCATACTGGCCCACGCACTGCCAGGTAAGTAATAACTTCGTATAAGGTATCCTATACGAAGTTATTCTCTCTGCCTATTGGGGTTACAAGACAGGTTTAAGGAGACCAATAGAAACTGGGCATGTGGAGACAGAGAAGACTCTTGGGTTTCTGATAGGCACTGACATAACTTCGTATAAGGTATCCTATACGAAGTTATTTTCCCTCCCTCAGGCTCTCCATCAAAGGCTGGTTCCTGCGACAGGGCCTGCTGGACACCAGCCTCACGG

**Mlh1**: CAAGGTCTACGCTTACCAGATGG

AGGGGTGGCTTCCTCATCCACTAGTGGAAGTGGCGACAAGGTCTACGCTTACCAGGTAAGTAGAAGTTCCTATTCtctagaaaGtATAGGAACTTCTCTCTCTGCCTATTGGGGTTACAAGACAGGTTTAAGGAGACCAATAGAAACTGGGCATGTGGAGACAGAGAAGACTCTTGGGTTTCTGATAGGCACTGACGAAGTTCCTATTCtctagaaaGtATAGGAACTTCTTTCCCTCCCTCAGATGGTCCGTACGGACTCCCGGGAGCAGAAGCTTGACGCCTTTCTGCAGCCTGTAAG

**Ace2**: GACGCTTGATGATCGGAGTCTGG

CTTTTATGAAGAACAGTCTAAGACTGCCCAAAGTTTCTCACTACAAGAAATCCAGGTAAGTAGAAGTTCCTATTCtctagaaaGtATAGGAACTTCTCTCTCTGCCTATTGGGGTTACAAGACAGGTTTAAGGAGACCAATAGAAACTGGGCATGTGGAGACAGAGAAGACTCTTGGGTTTCTGATAGGCACTGACGAAGTTCCTATTCtctagaaaGtATAGGAACTTCTTTCCCTCCCTCAGACTCCGATCATCAAGCGTCAACTACAGGCCCTTCAGCAAAGTGGGTCTTCAGCACT

**Usp42**: GGCGCTGGGCTCCAGAATTTGGG

TGAAAAGATTTGTCTTAAGTGGCAACAAAGTCATCGAGTTGGCGCTGGGCTCCAGGTAAGTAATAACTTCGTATAAGGTATCCTATACGAAGTTATTCTCTCTGCCTATTGGGGTTACAAGACAGGTTTAAGGAGACCAATAGAAACTGGGCATGTGGAGACAGAGAAGACTCTTGGGTTTCTGATAGGCACTGACATAACTTCGTATAAGGTATCCTATACGAAGTTATTTTCCCTCCCTCAGAATTTGGGCAACACCTGTTTTGCCAATGCCGCATTGCAGTGTCTGACTTACACGCC

**Sav1**: TTGCAGTTCTCAGGAAACTCTGG

TTCAAGTGCTACTGCTTTCTCAGCTTCTGGAGATGGTGTAGTTTCAAGAAACCAGGTAAGTAGAAGTTCCTATTCtctagaaaGtATAGGAACTTCTCTCTCTGCCTATTGGGGTTACAAGACAGGTTTAAGGAGACCAATAGAAACTGGGCATGTGGAGACAGAGAAGACTCTTGGGTTTCTGATAGGCACTGACGAAGTTCCTATTCtctagaaaGtATAGGAACTTCTTTCCCTCCCTCAGAGTTTCCTGAGAACTGCAATTCAAAGGACACCTCATGAAGTAATGAGAAGAGAAAG

**Rnf34**: CGGTTGGTATGTTTCTGAGGAGG

GCCCTCAGTTGATGCGACTAAAAGTGAAGGACCTGCGGCAGTATCTCCTCCTCAGGTAAGTAATAACTTCGTATAAGGTATCCTATACGAAGTTATTCTCTCTGCCTATTGGGGTTACAAGACAGGTTTAAGGAGACCAATAGAAACTGGGCATGTGGAGACAGAGAAGACTCTTGGGTTTCTGATAGGCACTGACATAACTTCGTATAAGGTATCCTATACGAAGTTATTTTCCCTCCCTCAGAAACATACCAACCGACACTTGTCGTGAGAAGGAAGACTTGGTGGATCTAGTACTGT

**Abi3**: TCTCAGGAGGGATGACTTTCTGG

AGCTAGAAGGGAGATCGGCACTTTGGCCACTGTTGTGCGGCTGCCCTCTAACCAGGTAAGTAATAACTTCGTATAAGGTATCCTATACGAAGTTATTCTCTCTGCCTATTGGGGTTACAAGACAGGTTTAAGGAGACCAATAGAAACTGGGCATGTGGAGACAGAGAAGACTCTTGGGTTTCTGATAGGCACTGACATAACTTCGTATAAGGTATCCTATACGAAGTTATTTTCCCTCCCTCAGAAAGTCATCCCTCCTGAGAGCCTGCCTTCCCTCACTCCCTACCACAGAAAACCCCT

**Lpar1**: GCTCACTGTGTTCCATTCTGTGG

TACAATGAGTCTATCGCCTTCTTTTATAACCGGAGTGGGAAATATCTAGCCACAGGTAAGTAATAACTTCGTATAAGGTATCCTATACGAAGTTATTCTCTCTGCCTATTGGGGTTACAAGACAGGTTTAAGGAGACCAATAGAAACTGGGCATGTGGAGACAGAGAAGACTCTTGGGTTTCTGATAGGCACTGACATAACTTCGTATAAGGTATCCTATACGAAGTTATTTTCCCTCCCTCAGAATGGAACACAGTGAGCAAGCTGGTGATGGGACTGGGCATCACTGTTTGCGTGTTC

**Tert**: GCTGTGCCTACCAGGTGTGTGGG

CCTGCTGGCACACTGTGCTCTTTATCTTCTGGTGCCCCCCAGCTGTGCCTACCAGGTAAGTAGAAGTTCCTATTCtctagaaaGtATAGGAACTTCTCTCTCTGCCTATTGGGGTTACAAGACAGGTTTAAGGAGACCAATAGAAACTGGGCATGTGGAGACAGAGAAGACTCTTGGGTTTCTGATAGGCACTGACGAAGTTCCTATTCtctagaaaGtATAGGAACTTCTTTCCCTCCCTCAGGTGTGTGGGTCTCCCCTGTACCAAATTTGTGCCACCACGGATATCTGGCCCTCTGT

**Zmpste24**: ACCAAACAAAGACAACCAGGAGG

AAGAGAATAGTTTTGTTTGACACTCTACTAGAAGAGTACTCTGTACCAAACAAAGGTAAGTAGAAGTTCCTATTCtctagaaaGtATAGGAACTTCTCTCTCTGCCTATTGGGGTTACAAGACAGGTTTAAGGAGACCAATAGAAACTGGGCATGTGGAGACAGAGAAGACTCTTGGGTTTCTGATAGGCACTGACGAAGTTCCTATTCtctagaaaGtATAGGAACTTCTTTCCCTCCCTCAGACAACCAGGAGGAGTCTGGCATGGAAGCCCGCAATGAAGGTGAAGGGGACAGTGAA

**Cdh12**: CACTCTTCGTGCTCAGGCAGTGG

TGCAATAAGAAGCCTGGATAGAGAAGAAAAACCTTTCTACACTCTTCGTGCTCAGGTAAGTAATAACTTCGTATAAGGTATCCTATACGAAGTTATTCTCTCTGCCTATTGGGGTTACAAGACAGGTTTAAGGAGACCAATAGAAACTGGGCATGTGGAGACAGAGAAGACTCTTGGGTTTCTGATAGGCACTGACATAACTTCGTATAAGGTATCCTATACGAAGTTATTTTCCCTCCCTCAGGCAGTGGACATAGAAACCAGGAAGCCACTGGAGCCTGAATCAGAGTTCATCATTAA

**RNAse4**: CATGTGGACCCTCAGGTGACAGG

GGGCTTGTACAGCCCTCCTATGGCCAGGATCGAATGTACCAACGGTTCCTTCGACAGCATGTGGACCCTCAGGTAAGTAATAACTTCGTATAAGGTATCCTATACGAAGTTATTCTCTCTGCCTATTGGGGTTACAAGACAGGTTTAAGGAGACCAATAGAAACTGGGCATGTGGAGACAGAGAAGACTCTTGGGTTTCTGATAGGCACTGACATAACTTCGTATAAGGTATCCTATACGAAGTTATTTTCCCTCCCTCAGGTGACAGGTGGCAATGACAACTACTGCAACGTGATGATGCAGAGACGGAAGATGACTTCTGTCCAGTGCA
